# BMPR2 Dosage Gates BMP9/10 Signaling Output in Pulmonary Artery Endothelium

**DOI:** 10.3390/cells15060492

**Published:** 2026-03-10

**Authors:** Kit-Yee Chu, Vijayalakshmi Thamilselvan, Amberly N. Crawford, Paul B. Yu, Erik Martinez-Hackert

**Affiliations:** 1 Department of Biochemistry and Molecular Biology, Michigan State University, 603 Wilson Road, East Lansing, MI 48824, USA; chukit@msu.edu (K.-Y.C.); crawf398@msu.edu (A.N.C.); 2 Cardiovascular Research Center, Massachusetts General Hospital, Harvard Medical School, 149 13th Street, Boston, MA 02129, USA; pbyu@mgh.harvard.edu

**Keywords:** BMPR2, BMP9/BMP10, ACVR2A, endothelial dysfunction, signal transduction, pulmonary arterial hypertension, bimagrumab

## Abstract

Pulmonary arterial hypertension (PAH) is characterized by dysfunction and remodeling of the pulmonary artery endothelium and smooth muscle. In heritable PAH, heterozygous loss-of-function mutations in the type II Bone Morphogenetic Protein (BMP) receptor gene (*BMPR2*) are the most common genetic cause. However, the mechanisms by which reduced BMPR2 levels alter endothelial signaling to drive PAH pathogenesis remain incompletely understood. To determine how BMPR2 levels govern signaling output and endothelial functional responses, we modulated BMPR2 expression in human pulmonary artery endothelial cells (PAECs) and assessed ligand-dependent SMAD1/5/8 signaling, proliferation, and caspase-3/7 activity. We found that BMP9 and BMP10 robustly activated SMAD1/5/8 signaling and promoted proliferation in PAECs, whereas the other ligands in this panel did not elicit a comparable signaling or proliferative response under these assay conditions. A moderate (~50%) reduction in BMPR2 protein levels (an in vitro approximation of haploinsufficiency) attenuated BMP9/10-induced SMAD1/5/8 activation, abolished proliferative responses, and was associated with a modest increase in caspase-3/7 activity, consistent with caspase pathway activation and early stress/injury signaling. Under BMPR2-limiting conditions, BMP9/10 responses became sensitive to Activin type II receptor blockade by bimagrumab, consistent with a context-dependent contribution of Activin type II receptors. Conversely, BMPR2 overexpression enhanced BMP9/10-dependent SMAD signaling and proliferation. Together, these findings support a receptor–dosage model where physiological BMPR2 expression is required to sustain homeostatic BMP9/10 signaling in pulmonary artery endothelium. This framework provides a basis for interpreting context-dependent pathway effects in PAH.

## 1. Introduction

Transforming Growth Factor-β (TGF-β) family signaling pathways regulate cellular differentiation, proliferation, and tissue homeostasis across metazoans [1,2]. In mammals, this family comprises over 30 ligands, including TGF-βs, Activins, Bone Morphogenetic Proteins (BMPs), and Growth and Differentiation Factors (GDFs) [3]. These ligands signal through heterotetrameric complexes composed of type I and type II serine/threonine kinase receptors. Ligand binding enables type II receptor–mediated activation of the type I receptors, leading to Small body size (Sma)/Mothers Against Decapentaplegic (MAD) (SMAD) phosphorylation and downstream transcriptional regulation [1,4]. In the vasculature, tight control of these pathways is required to maintain endothelial and smooth muscle homeostasis, and dysregulation contributes to pathological tissue remodeling in vascular disease.

Pulmonary arterial hypertension (PAH) is a progressive and life-threatening condition characterized by remodeling and narrowing of small pulmonary arteries. These changes increase pulmonary vascular resistance, leading to right ventricular failure and ultimately premature death [5,6]. A major genetic cause of PAH is heterozygous loss-of-function mutations in the *BMPR2* gene, which encodes the type II BMP receptor [7,8,9]. *BMPR2* mutations are present in approximately 70–80% of heritable PAH (HPAH) cases and 10–20% of idiopathic PAH (IPAH) cases [10]. PAH-associated pathogenic variants have also been reported in other components of the endothelial BMP signaling axis, including *ACVRL1* (encoding the type I receptor ALK1), *ENG* (the co-receptor endoglin), and the ligand genes *BMP9* (*GDF2*) and *BMP10* [11,12,13,14]. Functionally, these mutations reduce signaling capacity through the ALK1–BMPR2–SMAD1/5/8 pathway [15]. As reduced BMPR2 levels have also been reported in pulmonary vascular tissue from PAH patients without BMPR2 mutations [16,17], these observations suggest that BMPR2 insufficiency and impaired signaling through this axis are common features in a substantial subset of PAH.

BMPR2 insufficiency is associated with altered vascular cell behaviors relevant to pulmonary vascular remodeling, including changes in proliferation, survival, and differentiation [16,17,18,19,20,21,22,23]. These effects are cell-type specific. In pulmonary artery smooth muscle cells (PASMCs), reduced BMPR2 signaling has been linked to hyperproliferation and apoptosis resistance consistent with medial hypertrophy [18,20,21]. In contrast, pulmonary artery endothelial cells (PAECs) exhibit impaired homeostasis under BMPR2 deficiency, including increased apoptosis and defective repair, with the latter emergence of apoptosis-resistant, hyperproliferative subpopulations in some models [19,24,25,26,27]. Together, these divergent responses are thought to contribute to characteristic PAH lesions, including arterial wall thickening and complex vascular remodeling [23,28,29,30]. Despite these well-described phenotypes, it remains unclear how quantitative reductions in BMPR2 abundance reshape endothelial signaling downstream of its physiological ligands, or how such changes are translated into altered proliferative and survival responses [26,31,32,33,34,35,36]. This knowledge gap underscores the need to define how BMPR2 dosage modulates ligand-dependent signaling output in pulmonary artery endothelium.

Among TGF-β superfamily ligands, BMP9 and BMP10 represent the principal circulating inputs into ALK1–BMPR2 signaling in pulmonary artery endothelium, making them the most physiologically relevant ligands for probing the consequences of BMPR2 insufficiency. Consistent with this central role, BMP9/10 signaling has been extensively manipulated in experimental models of pulmonary hypertension, yielding strongly context-dependent outcomes often described as “Janus” effects [37,38,39,40]. In some settings, BMP9 administration improves endothelial function and reverses disease features [37], whereas in others, inhibition of BMP9 signaling is beneficial [38,39]. Notably, this apparent paradox extends to the clinic, as evidenced by the efficacy of sotatercept, an ACVR2A-based ligand trap that inhibits ligands within the BMP9/10–Activin signaling axis and confers clinical benefit in PAH [41,42]. Together, these observations indicate that ligand availability alone does not determine biological outcome, raising the possibility that endothelial receptor context contributes to the diverse effects of BMP9/10 signaling.

To address this knowledge gap, we modulated BMPR2 expression in primary human PAECs. This approach enabled us to examine how receptor dosage shapes BMP9/10 signaling and endothelial functional responses. We assessed BMP9/10-dependent SMAD1/5/8 activation together with proliferation and caspase-3/7 activity and probed the contribution of Activin type II receptors using the neutralizing antibody bimagrumab. These experiments support the interpretation that physiological BMPR2 expression is required for robust BMP9/10-induced signaling and proliferative responses, whereas moderate reductions modeling haploinsufficiency impair signaling output and alter downstream functional outcomes. Under BMPR2-limiting conditions, BMP9/10 responses also become sensitive to blockade of Activin type II receptors, indicating context-dependent receptor usage when BMPR2 availability is reduced. Collectively, these findings support a receptor–dosage framework in which BMPR2 expression levels govern BMP9/10 signaling outcomes in pulmonary artery endothelium, providing a rationale for the context-dependent effects of BMP9/10 pathway modulation seen in experimental models.

## 2. Materials and Methods

Growth Factors and Reagents: Human TGF-β1 (P01137), BMP7 (P18075), BMP9 (Q9UK05), and GDF11 (O95390) were obtained from R&D Systems (Minneapolis, MN, USA). Activin A (INHBA; P08476), Activin B (INHBB; Q6NXH1), and BMP10 (O95393) were produced in-house using stably transfected CHO cells as previously described. Briefly, Fc-tagged Activin A and His-tagged BMP10 were purified from conditioned medium by affinity chromatography (ÄKTA Pure, Cytiva, Marlborough, MA, USA). Growth factor moieties were separated from their pro-domains using reversed-phase chromatography (Resource RPC, Cytiva) under acidic conditions. All growth factors were lyophilized and stored at −80 °C.

Receptor-Fc Fusions and Antibodies: Human BMPR2 cDNA (Q13873) and synthetic genes encoding human ACVR2A (P27037) and ACVR2B (Q13705) were fused to a human IgG1-Fc domain. Constructs included the signal peptide and extracellular domains of ACVR2A (aa 1–120), ACVR2B (aa 1–120), and BMPR2 (aa 1–136). Fusion proteins and Bimagrumab were expressed in stably transfected CHO cells and purified from conditioned medium using Protein A affinity chromatography followed by size-exclusion chromatography. Purified proteins were stored in phosphate-buffered saline (pH 7.5) at −80 °C. Purity was confirmed by SDS-PAGE.

Small-Molecule Inhibitors: LDN-193189 was purchased from BioVision (Milpitas, CA, USA) and reconstituted in DMSO according to the manufacturer’s instructions. Equivalent concentrations of DMSO were included in all vehicle control conditions.

Cell Lines and Culture: Human primary pulmonary artery endothelial cells (PAECs; ATCC PCS-100-022), pulmonary artery smooth muscle cells (PASMCs; ATCC PCS-100-023), and HEK293 cells (ATCC CRL-1573) were obtained from the American Type Culture Collection (Manassas, VA, USA). Additional primary PAECs and PASMCs were obtained from Lonza (Walkersville, MD, USA). PAECs were cultured in Vascular Cell Basal Medium (ATCC PCS-100-030) supplemented with the Endothelial Cell Growth Kit-VEGF (ATCC PCS-100-041) and 1% penicillin/streptomycin. PASMCs were cultured in smooth muscle cell basal medium supplemented with manufacturer-recommended growth supplements. HEK293 cells were maintained in Eagle’s Minimum Essential Medium supplemented with 10% fetal bovine serum and 1% penicillin/streptomycin. All experiments were performed using cells between passages 4 and 8. Data shown are representative of experiments performed in PAECs from both ATCC and Lonza.

Luciferase Reporter Assays: SMAD1/5/8-responsive (BRE) or SMAD2/3-responsive (SBE) reporter cells were seeded in 96-well plates and cultured overnight. Cells were treated in serum-free medium with 5.0 nM growth factors and titrated concentrations of receptor-Fc fusion proteins or Bimagrumab (0–300 nM). For receptor overexpression studies, cells were transfected with myc-tagged ALK1 and HA-tagged BMPR2, ACVR2A, or ACVR2B plasmids using TransIT-293 reagent Mirus Bio (Madison, WI, USA). Firefly luciferase activity was measured after 16 h using a FLUOstar Omega plate reader BMG Labtech (Ortenberg, Germany).

Cell Proliferation Assays: PAEC proliferation was quantified using a BrdU incorporation assay. Cells were serum-restricted overnight and treated with growth factors (0.8 nM) in the presence or absence of inhibitors as indicated. BrdU incorporation was measured spectrophotometrically at 450 nm and normalized to untreated controls.

Caspase-3/7 Assays: Caspase-3/7 activity was measured using the Caspase-Glo^®^ 3/7 Assay (Promega, Madison, WI, USA). PAECs were treated with BMP9 or BMP10 (0.8 nM) for 24 h under control or BMPR2 knockdown conditions. Caspase-3/7 enzymatic activity was quantified as a surrogate marker of effector caspase pathway engagement and stress-associated signaling; this assay does not on its own establish apoptotic commitment.

siRNA Transfection: PAECs were transfected with pooled BMPR2-targeting siRNA (Dharmacon ON-TARGETplus SMARTpool, (Lafayette, CO, USA)) or non-targeting control siRNA (Dharmacon D-001810-02-05) using DharmaFECT 1 reagent. Cells were returned to complete medium after 4 h and used for signaling or functional assays as described.

Immunoblotting: Protein lysates were prepared in RIPA buffer (Thermo Fisher Scientific, Waltham, MA, USA) containing Protease Arrest and Phosphatase Arrest protease and phosphatase inhibitor cocktails (G-Biosciences, St. Louis, MO, USA). Equal amounts of protein were resolved by SDS-PAGE and transferred to PVDF membranes (Cytiva, Marlborough, MA, USA). Primary antibodies included anti-phospho-SMAD1/5/8 and anti-phospho-SMAD2/3 (Cell Signaling Technology, Danvers, MA, USA), anti-BMPR2 (clone 1F12, catalog no. ab130206; Abcam, Cambridge, UK), anti-ACVR2A (catalog no. AF340-SP; R&D Systems, Minneapolis, MN, USA), and anti-ACVR2B (catalog no. AF339-SP; R&D Systems). β-actin and GAPDH served as loading controls as indicated and were detected using β-Actin (13E5) Rabbit mAb (catalog no. #4970; Cell Signaling Technology) and GAPDH (D16H11) Rabbit mAb (catalog no. #5174; Cell Signaling Technology), respectively. Signals were detected by chemiluminescence and quantified by densitometry using ImageJ/Fiji 1.53t (National Institutes of Health, Bethesda, MD, USA).

Statistical Analysis: Experiments were performed using PAECs and PASMCs from two commercial sources (Lonza and ATCC) or HEK293 cells (ATCC). For each experiment, cells were plated in triplicate from the same cell batch. Within each experiment, data were analyzed by two-tailed unpaired *t*-tests or one- or two-way ANOVA with post hoc corrections (Dunnett’s, Šidák’s, or Tukey’s) as appropriate, using *n* = 3 replicate wells per condition unless noted. *p* < 0.05 was considered statistically significant. Data are mean ± SD of *n* = 3 technical replicate wells. Experiments were repeated independently on multiple occasions with similar results.

Use of Generative Artificial Intelligence: Generative artificial intelligence tools were used for assistance with language editing and clarity of written text. These tools were not used for data generation, experimental design, data analysis, interpretation, or the creation or modification of data figures.

## 3. Results

### 3.1. BMP9 and BMP10 Emerge as Specific Mitogens for Pulmonary Artery Endothelial Cells

To elucidate the functional specificity of TGF-β family ligands in pulmonary arterial vascular biology, we first screened a panel of TGF-β family ligands for their effects on the two major pulmonary artery cell types, endothelial cells (PAECs) and smooth muscle cells (PASMCs), under serum-starved conditions [43,44]. This systematic approach used a standardized ligand concentration of 0.8 nM to enable direct receptor–dosage comparisons under serum-restricted conditions. This concentration lies within the reported high-picomolar to low-nanomolar biologically active range for circulating BMP9/10, recognizing that precise quantification varies depending on circulating form and detection method. The panel included BMP9, BMP10, Activin A, Activin B, GDF11, BMP7, and TGF-β1, allowing us to assess functional specificity in a controlled setting [45,46,47,48,49,50].

**Figure 1:**
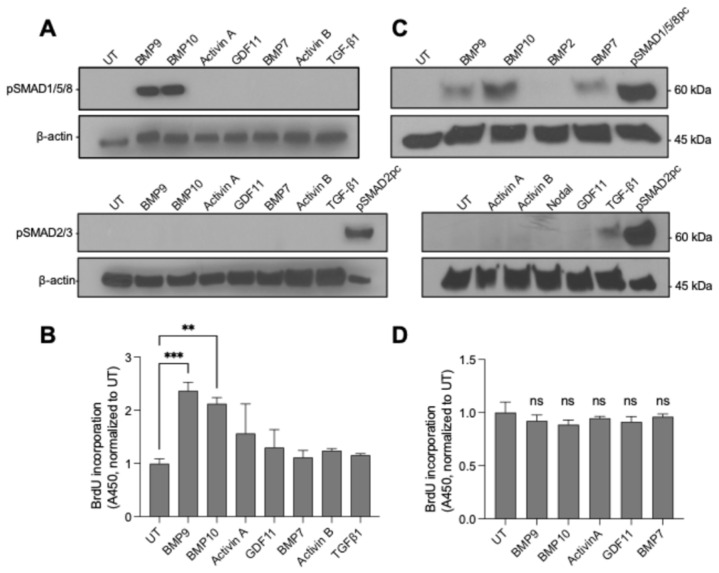
BMP9 and BMP10 selectively activate SMAD1/5/8 signaling and induce proliferation in pulmonary artery endothelial cells but not pulmonary artery smooth muscle cells. (**A**) Western blot analysis of phosphorylated SMAD1/5/8 and SMAD2/3 in PAECs treated with the indicated TGF-β superfamily ligands (0.8 nM) or untreated control (UT); β-actin serves as a loading control. (**B**) PAEC proliferation measured by BrdU incorporation following ligand treatment (0.8 nM), normalized to UT. (**C**) Western blot analysis of phosphorylated SMAD1/5/8 and SMAD2/3 in PASMCs treated with the indicated ligands (0.8 nM); β-actin serves as a loading control. (**D**) PASMC proliferation measured by BrdU incorporation following ligand treatment (0.8 nM), normalized to UT. Data are shown as mean ± SD (*n* = 3 replicate wells). Statistical significance was assessed by one-way ANOVA with Dunnett’s multiple-comparisons test (each ligand vs. UT). Statistical significance was assessed by one-way ANOVA with Dunnett’s multiple-comparisons test (each ligand vs. UT). ** *p* < 0.01, *** *p* < 0.001; ns, not significant.

Our signaling screen in PAECs revealed a high degree of specificity among TGF-β family members. Among the ligands tested, BMP9 and BMP10 induced robust SMAD1/5/8 phosphorylation, whereas SMAD2/3 phosphorylation was not detected under these conditions (Figure 1A). This specific signaling response was associated with a distinct functional outcome. Treatment with either BMP9 or BMP10 led to enhanced PAEC proliferation, as quantified by BrdU incorporation (Figure 1B). Other ligands tested did not elicit comparable signaling or proliferative responses under these conditions, underscoring the unique roles of BMP9 and BMP10 as regulators of PAEC growth.

In contrast, PASMCs exhibited a distinct response pattern. Although BMP9, BMP10, and BMP7 activated SMAD1/5/8 signaling to varying degrees (Figure 1C), none increased PASMC proliferation relative to untreated controls (BrdU assay, Figure 1D). Thus, while PASMCs retain the capacity to respond to BMP9/10 at the signaling level, their proliferation is not directly governed by this pathway.

Having established BMP9 and BMP10 as the only ligands in this panel that couple SMAD1/5/8 activation to a proliferative response in PAECs, we next investigated the signaling pathway mediating this mitogenic effect.

### 3.2. The Mitogenic Effect of BMP9 and BMP10 Is Mediated by SMAD1/5/8 Signaling

After identifying BMP9/10 as specific PAEC mitogens, we sought to determine the mechanism underlying this proliferation. Our phosphorylation data implicated SMAD1/5/8 as the mediator of BMP9/10’s effects. However, BMP receptors can also activate non-SMAD pathways such as Akt, MAPK, and PI3K [51,52,53,54]. To isolate the specific contribution of SMAD1/5/8 signaling to the mitogenic response, we employed LDN-193189, a widely used and well-characterized small molecule inhibitor of BMP type I receptor kinases [55].

**Figure 2:**
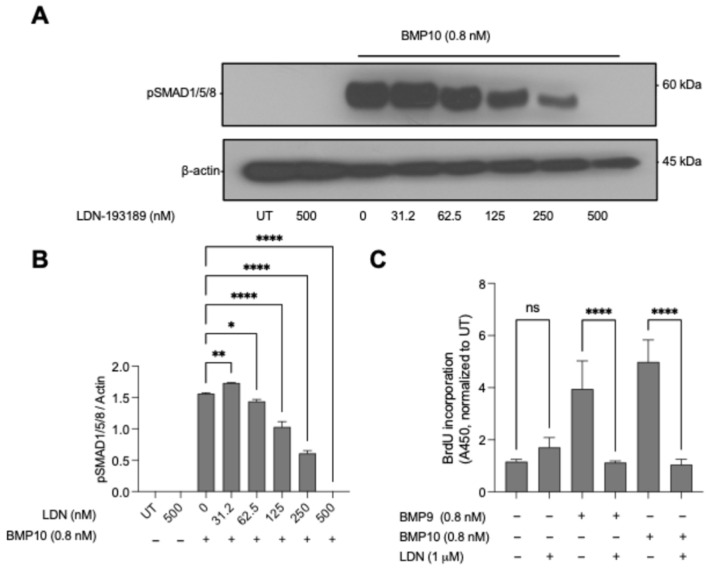
BMP9- and BMP10-induced PAEC proliferation requires SMAD1/5/8 signaling. (**A**) Dose-dependent inhibition of BMP10 (0.8 nM)-induced SMAD1/5/8 phosphorylation in PAECs by LDN-193189 (0–500 nM); β-actin serves as a loading control. (**B**) Densitometric quantification of (**A**) (*n* = 2 independent blots). (**C**) PAEC proliferation measured by BrdU incorporation following BMP9 or BMP10 treatment (0.8 nM) ± LDN-193189 (1 µM), normalized to UT. Data are shown as mean ± SD (*n* = 3 replicate wells). Statistical significance was assessed by one-way ANOVA with Dunnett’s multiple-comparisons test for (**B**) (each dose vs. 0 nM LDN) and by one-way ANOVA with Šidák’s multiple-comparisons test for (**C**) (comparisons as indicated). Statistical significance was assessed by one-way ANOVA with Dunnett’s multiple-comparisons test (each ligand vs. UT). * *p* < 0.05, ** *p* < 0.01, **** *p* < 0.0001; ns, not significant.

Dose–response analysis revealed that LDN-193189 effectively blocked BMP10-induced SMAD1/5/8 phosphorylation in PAECs in a concentration-dependent manner, with near-complete inhibition achieved at 500 nM (Figure 2A,B). This dose-dependent suppression of SMAD1/5/8 activation provided a clear pharmacological tool to interrogate pathway specificity. Thus, we used 1 µM LDN-193189 in subsequent functional assays, a concentration based on the dose–response in Figure 2A,B that robustly suppresses BMP type I kinase activity under our conditions [56]. Using BrdU assays, we found that 1 µM LDN-193189 completely abolished BMP9- and BMP10-induced PAEC proliferation (Figure 2C). This complete reversal of the mitogenic effect indicates that BMP type I receptor kinase activity is required for the mitogenic response, consistent with SMAD1/5/8 as the principal effector pathway. These findings indicate that despite the known complexity of BMP receptor signaling networks, the mitogenic response of PAECs to BMP9 and BMP10 is channeled through the SMAD1/5/8 effector pathway.

### 3.3. BMPR2 Is the Primary Receptor for BMP9 and BMP10 in Pulmonary Artery Endothelium

**Figure 3:**
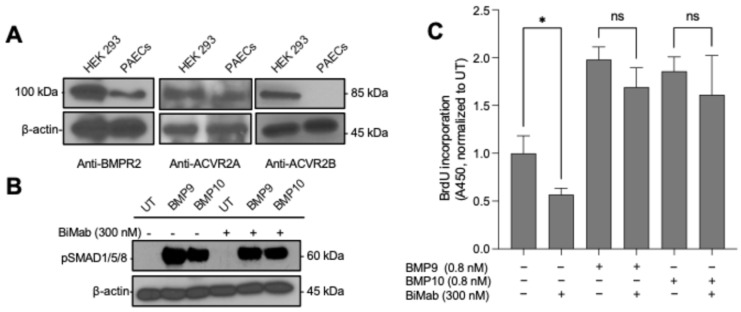
BMPR2 is the dominant type II receptor mediating BMP9/10 signaling in PAECs under basal conditions. (**A**) Immunoblot analysis of type II receptor expression (BMPR2, ACVR2A, ACVR2B) in HEK293 cells and PAECs; β-actin serves as a loading control. (**B**) Immunoblot analysis of SMAD1/5/8 phosphorylation in PAECs treated with BMP9 or BMP10 (0.8 nM) ± bimagrumab (BiMab; 300 nM); β-actin serves as a loading control. (**C**) PAEC proliferation measured by BrdU incorporation following BMP9 or BMP10 treatment (0.8 nM) ± bimagrumab (300 nM), normalized to UT. Data are shown as mean ± SD (*n* = 3 replicate wells). Statistical significance was assessed by one-way ANOVA with Šidák’s multiple-comparisons test (comparisons as indicated). Statistical significance was assessed by one-way ANOVA with Dunnett’s multiple-comparisons test (each ligand vs. UT). * *p* < 0.05; ns, not significant.

BMP9 and BMP10 are known to signal via the type I receptor ALK1. However, identifying which type II receptor they predominantly use in PAECs is critical to understanding their endothelial effects. TGF-β family ligands often bind multiple receptors, with BMP9 and BMP10 capable of binding three potential type II receptors: BMPR2, ACVR2A, and ACVR2B [57,58,59]. Our initial characterization by Western blot revealed that PAECs endogenously express high levels of BMPR2 and ACVR2A, but not ACVR2B (Figure 3A), narrowing the functional candidates for our studies.

To assess the contribution of the type II activin receptors ACVR2A and ACVR2B (ACVR2) to BMP9/10 signaling under basal conditions, PAECs were treated with BMP9 or BMP10 in the presence or absence of the ACVR2-blocking antibody Bimagrumab (BiMab) [60]. Despite the confirmed expression of ACVR2A in PAECs, its functional contribution to BMP9/10-induced proliferation was not detected in our assays when BMPR2 is at normal levels. Specifically, 300 nM Bimagrumab did not affect BMP9/10-induced SMAD1/5/8 phosphorylation (Figure 3B), indicating that canonical BMP9/10 signaling in PAECs is not measurably ACVR2-dependent when BMPR2 expression is intact. Consistent with these signaling data, BMP9- and BMP10-induced proliferation, measured by BrdU incorporation, was not reduced by ACVR2 blockade (Figure 3C). Bimagrumab did not alter baseline SMAD phosphorylation or BrdU incorporation under vehicle conditions, indicating the lack of effect reflects BMP9/10-specific signaling in PAECs. Together, these results support BMPR2 as the dominant type II receptor mediating BMP9/10-dependent SMAD1/5/8 activation and proliferative responses in PAECs under basal conditions.

### 3.4. Comprehensive Receptor Usage Analysis in HEK293 Cells

In PAECs expressing BMPR2 at endogenous levels, bimagrumab did not measurably inhibit BMP9/10-induced SMAD1/5/8 signaling or proliferation (Figure 3B,C), even though ACVR2A is detectable and ACVR2B is not (Figure 3A). To determine whether this apparent BMPR2 dominance reflects a PAEC-specific expression context or a broader feature of ALK1 ligand–type II receptor pairing, we evaluated BMP10 receptor usage in HEK293 reporter cells, which express BMPR2, ACVR2A, and ACVR2B (Figure 3A).

**Figure 4:**
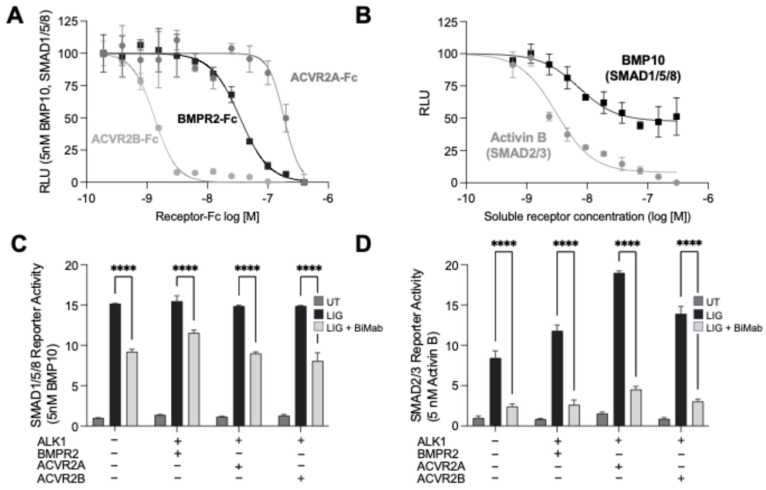
BMP10 can engage multiple type II receptors, whereas Activin B signaling is fully Activin type II receptor–dependent in HEK293 cells. (**A**) Dose-dependent inhibition of BMP10-induced SMAD1/5/8 reporter activity in HEK293 cells by soluble receptor ectodomain–Fc fusions (BMPR2-Fc, ACVR2A-Fc, ACVR2B-Fc); BMP10 was used at 5 nM. (**B**) Differential inhibition of BMP10-induced SMAD1/5/8 reporter activity and Activin B–induced SMAD2/3 reporter activity by bimagrumab (0–300 nM). Reporter output in (**A**,**B**) is reported as relative luminescence units (RLU), background-subtracted using untreated control (UT) and normalized to the ligand-only condition (set to 100) within each experiment. (**C**) SMAD1/5/8 reporter activity in HEK293 cells co-expressing ALK1 and the indicated type II receptor, stimulated with BMP10 (5 nM) ± bimagrumab (300 nM). (**D**) SMAD2/3 reporter activity under the conditions in (**C**), stimulated with Activin B (5 nM) ± bimagrumab (300 nM). Data are shown as mean ± SD (*n* = 3 replicate wells). Statistical significance was assessed by two-way ANOVA with Tukey’s multiple-comparisons test (comparisons as indicated). Statistical significance was assessed by one-way ANOVA with Dunnett’s multiple-comparisons test (each ligand vs. UT). **** *p* < 0.0001.

To determine whether the lack of ACVR2 involvement in PAECs reflects an intrinsic inability of BMP10 to engage ACVR2 ectodomains, we first tested ectodomain-level compatibility using soluble type II receptor ectodomain–Fc fusions as ligand traps (BMPR2-Fc, ACVR2A-Fc, ACVR2B-Fc). Each trap inhibited BMP10-induced SMAD1/5/8 reporter activity in a dose-dependent manner (Figure 4A), demonstrating that BMP10 can be intercepted in solution by all three type II receptor ectodomains. The traps differed in apparent neutralization potency (Table 1), indicating unequal binding efficiencies in this format. Although the soluble-trap assay does not recapitulate membrane complex assembly, the rank-order differences are consistent with ectodomain-level interaction preferences that could bias ligand partitioning among competing type II receptors at the cell surface.

We next asked which type II receptors contribute to BMP10 signaling when multiple candidates are present by using bimagrumab, which blocks ligand binding to ACVR2A/ACVR2B (ACVR2). As an internal control for bimagrumab activity, Activin B–induced SMAD2/3 reporter output was potently inhibited, with near-complete maximal inhibition (Figure 4B; Table 1). By contrast, bimagrumab produced only partial inhibition of BMP10-induced SMAD1/5/8 reporter output, plateauing with a substantial residual signal (Figure 4B; Table 1). This pattern indicates that in HEK293 cells a fraction of BMP10 signaling is ACVR2-dependent, but a large bimagrumab-resistant component remains, consistent with BMPR2-supported signaling that bimagrumab cannot block.

Finally, we tested whether type II receptor abundance biases BMP10 signaling downstream of ALK1 by co-expressing ALK1 with individual type II receptors and examining the effect of bimagrumab. BMP10 induced robust SMAD1/5/8 reporter activity across conditions (Figure 4C). Addition of bimagrumab reduced BMP10 reporter output, with the largest suppression observed when ACVR2A or ACVR2B was co-expressed and a higher residual signal maintained when BMPR2 was co-expressed (Figure 4C), consistent with BMPR2 supporting a bimagrumab-resistant component of BMP10 signaling. In parallel, Activin B–induced SMAD2/3 reporter activity was enhanced by ACVR2A and ACVR2B co-expression and remained strongly bimagrumab-sensitive under the same transfection conditions (Figure 4D), confirming effective ACVR2 blockade. Together, these experiments show that BMP10 can utilize ACVR2 receptors, but that BMPR2 availability shifts signaling toward a bimagrumab-resistant, canonical output.

### 3.5. BMPR2 Dosage Constrains BMP9/10 Signaling Sufficiency and PAEC Functional Output

**Figure 5:**
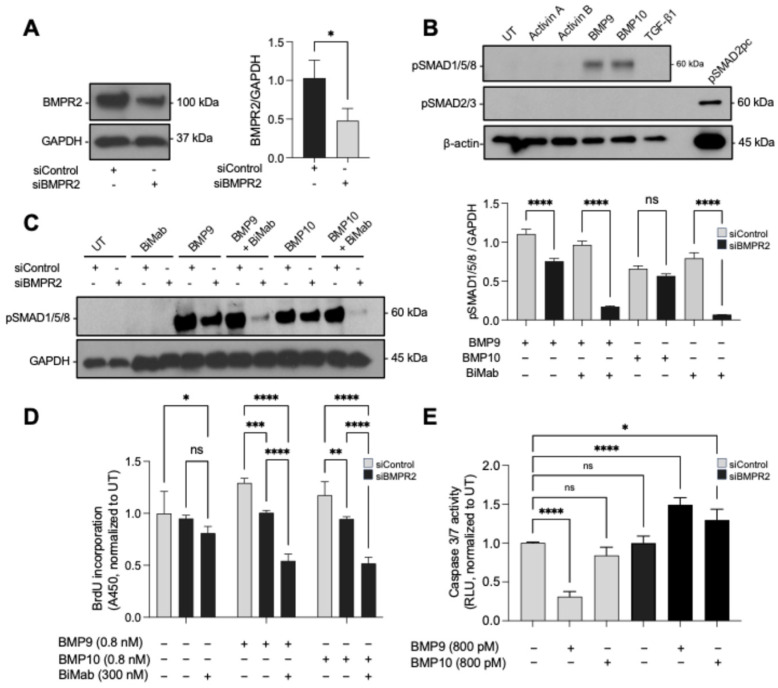
BMPR2 knockdown attenuates BMP9/10 signaling, abolishes proliferation, and confers sensitivity to Activin type II receptor blockade. (**A**) Immunoblot confirmation of BMPR2 knockdown in PAECs transfected with BMPR2-targeting siRNA (siBMPR2) or non-targeting control siRNA (siControl); GAPDH serves as a loading control; densitometric quantification shown. Statistical significance for densitometry was assessed by unpaired *t*-test (*n* = 3). (**B**) Immunoblot analysis of SMAD phosphorylation in siControl and siBMPR2 PAECs treated with the indicated ligands (0.8 nM). (**C**) SMAD1/5/8 phosphorylation in siControl and siBMPR2 PAECs treated with BMP9 or BMP10 (0.8 nM) ± bimagrumab (300 nM). The bar graph shows densitometric analysis (mean ± SD, *n* = 2 independent blots) of pSMAD1/5/8 normalized to GAPDH. Statistical significance for densitometry was assessed by one-way ANOVA with Šidák’s multiple-comparisons test (comparisons as indicated). (**D**) PAEC proliferation measured by BrdU incorporation under the conditions in (**C**), normalized to UT. Statistical significance was assessed by two-way ANOVA with Tukey’s multiple-comparisons test. (**E**) Caspase-3/7 activity measured under the conditions in (**C**), normalized to UT (surrogate marker of caspase pathway activation). Statistical significance was assessed by one-way ANOVA with Dunnett’s multiple-comparisons test (each condition vs. UT). For (**D**,**E**), data are shown as mean ± SD (*n* = 3 replicate wells). “pSMAD2pc” denotes a pSMAD2/3 positive control generated by inducing SMAD2/3 phosphorylation in HEK293 cells (e.g., TGF-β1–treated), included to verify pSMAD2/3 detection. Statistical significance was assessed by one-way ANOVA with Dunnett’s multiple-comparisons test (each ligand vs. UT). * *p* < 0.05, ** *p* < 0.01, *** *p* < 0.001, **** *p* < 0.0001; ns, not significant.

Heterozygous mutations in *BMPR2* are the most common genetic cause of familial PAH, leading to haploinsufficiency, reduced BMPR2 protein levels, and compromised pulmonary vascular remodeling [22,28,61]. To model reduced BMPR2 expression, PAECs were transfected with BMPR2-targeting siRNA, resulting in approximately 50% reduction in BMPR2 protein levels (Figure 5A). This moderate reduction in BMPR2 did not alter ligand specificity, as both control and knockdown cells retained their exclusive responsiveness to BMP9 and BMP10 (Figure 5B). The preserved ligand specificity in *BMPR2*-knockdown PAECs supports the interpretation that the functional consequences of BMPR2 reduction are expressed within the BMP9/10-responsive axis rather than reflecting generalized activation of non-BMP9/10 ligands under these conditions.

While ligand specificity remained unchanged, *BMPR2* knockdown altered downstream signaling and cellular outcomes. Specifically, *BMPR2* knockdown led to a marked reduction in BMP9- and BMP10-induced SMAD1/5/8 phosphorylation (Figure 5C) and abolished the proliferative response to both ligands (Figure 5D). Notably, under conditions of BMPR2 insufficiency, residual BMP9/10-induced SMAD1/5/8 phosphorylation became sensitive to ACVR2 blockade with bimagrumab (Figure 5C), in contrast to the BMPR2-replete state. This is consistent with ACVR2 contributing to remaining canonical signaling, although this signaling is insufficient to support a proliferative response.

BMPR2 knockdown was also associated with increased caspase-3/7 activity (Figure 5E), which we interpret as evidence of effector caspase pathway engagement and early cellular stress under BMPR2-limiting conditions rather than definitive apoptotic commitment. Notably, BMP9 elicited a quantitatively greater increase in caspase-3/7 activity than BMP10 under BMPR2 knockdown. This likely reflects differences in signaling amplitude and/or persistence rather than distinct qualitative signaling programs, as signaling kinetics were not directly assessed here.

### 3.6. Increasing BMPR2 Levels Enhances BMP9/10-Induced Proliferation

**Figure 6:**
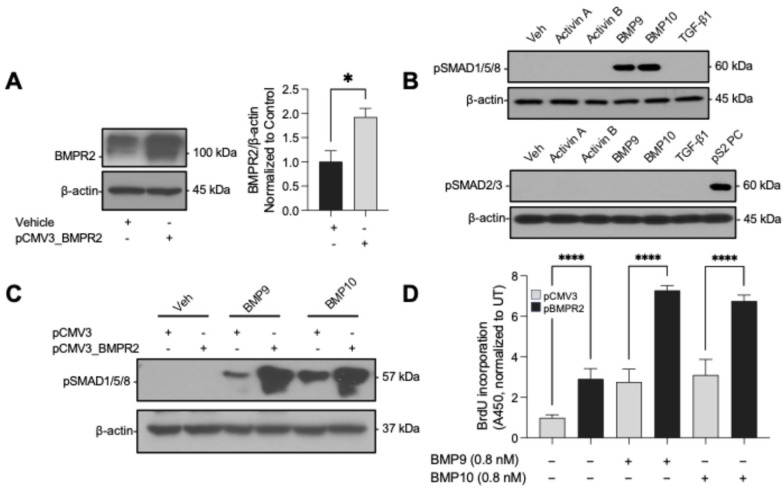
BMPR2 overexpression enhances BMP9/10-dependent SMAD1/5/8 signaling and proliferation in PAECs. (**A**) Immunoblot confirmation of BMPR2 overexpression in PAECs transfected with BMPR2 expression vector or empty vector control; β-actin serves as a loading control; densitometric quantification shown (*n* = 3). Statistical significance for densitometry was assessed by unpaired *t*-test. (**B**) Immunoblot analysis of SMAD phosphorylation in vector control and BMPR2-overexpressing PAECs treated with the indicated ligands (0.8 nM). (**C**) Immunoblot analysis of SMAD1/5/8 phosphorylation in vector control and BMPR2-overexpressing PAECs treated with BMP9 or BMP10 (0.8 nM); GAPDH serves as a loading control. (**D**) PAEC proliferation measured by BrdU incorporation under the conditions in (**C**), normalized to UT. Data are shown as mean ± SD (*n* = 3 replicate wells). Statistical significance was assessed by one-way ANOVA with Šidák’s multiple-comparisons test (comparisons as indicated). Statistical significance was assessed by one-way ANOVA with Dunnett’s multiple-comparisons test (each ligand vs. UT). * *p* < 0.05, **** *p* < 0.0001.

To complement our knockdown experiments and directly test whether BMPR2 levels govern the magnitude of BMP9/BMP10 responses, we investigated whether BMPR2 overexpression could enhance the cellular response in PAECs. Transfection with a BMPR2 expression vector resulted in an approximately 2-fold increase in receptor levels (Figure 6A), supporting a gain-of-function model that mirrors our loss-of-function studies.

Importantly, this overexpression maintained the pathway specificity observed previously. Enhanced BMPR2 levels did not non-specifically sensitize PAECs to other TGF-β family ligands, as evidenced by the absence of signaling responses beyond BMP9 and BMP10 (Figure 6B). This specificity underscores that BMPR2 modulation selectively affects BMP9/BMP10 signaling, rather than causing broad pathway dysregulation.

The functional consequences of BMPR2 overexpression were evident. In PAECs transfected with a BMPR2 expression vector, treatment with 0.8 nM BMP9 or BMP10 led to an increase in SMAD1/5/8 phosphorylation compared to control-transfected cells (Figure 6C), indicating that elevated receptor abundance enhances ligand-induced signaling. This increased signaling was accompanied by a nearly 3-fold increase in the proliferative response of BMPR2-overexpressing PAECs to both ligands (Figure 6D), underscoring the direct relationship between BMPR2 levels and functional cellular outcomes.

**Figure 7:**
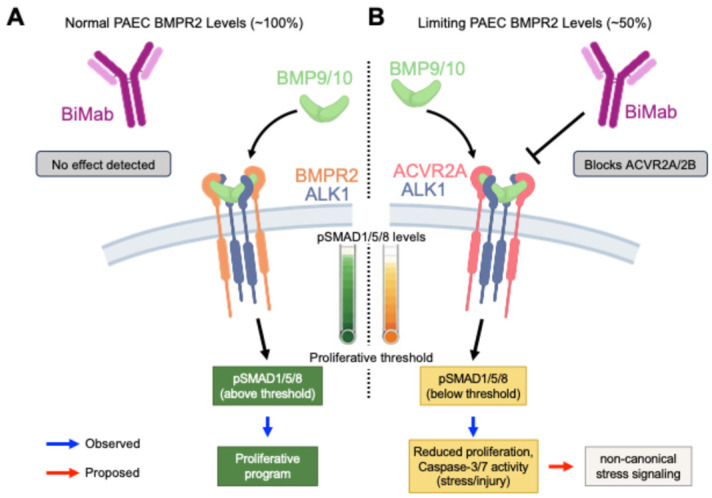
BMPR2 dosage-dependent model for BMP9/10 signaling output in pulmonary artery endothelial cells. Schematic illustrating how BMPR2 abundance constrains BMP9/10 (ALK1-dependent) canonical signaling output and downstream cellular programs in PAECs. (**A**) BMPR2-sufficient (~100%) state: BMP9/10 predominantly signal through ALK1–BMPR2 complexes, generating pSMAD1/5/8 output consistent with a threshold-like requirement for proliferation; bimagrumab (BiMab) produces no effect detected under BMPR2-replete conditions. (**B**) BMPR2-limiting (~50%) state: Reduced BMPR2 attenuates BMP9/10-induced canonical output and is associated with reduced proliferation and increased caspase-3/7 activity consistent with stress/injury. Under BMPR2-limiting conditions, residual canonical output becomes bimagrumab-sensitive, consistent with context-dependent contribution of Activin type II receptors (predominantly ACVR2A in PAECs; see Table 1 for BMP10 affinity comparisons) to the remaining pSMAD1/5/8 signal. A putative non-canonical stress-signaling arm is shown as a proposed intermediate. Solid arrows denote observed relationships; dashed arrows and dashed-outline boxes denote proposed steps. Node shading and output gauges depict relative canonical signaling output.

These gain-of-function experiments indicate that BMPR2 abundance is a rate-limiting factor that dictates both the sensitivity and magnitude of PAEC proliferative responses to BMP9 and BMP10. The proportional relationship between receptor levels and functional outcomes suggests that even modest increases in BMPR2 expression could significantly enhance the proliferative capacity of pulmonary artery endothelium. These findings are summarized schematically in a receptor–dosage model illustrating how BMPR2 expression level shifts BMP9/10 signaling outcomes in PAECs (Figure 7).

## 4. Discussion

A receptor–dosage framework for endothelial BMP9/10 signaling. BMP9 and BMP10 are circulating vascular ligands that regulate endothelial behavior through ALK1-containing receptor complexes, with signaling output critically dependent on the availability of type II receptors. Disruption of this axis is a well-recognized feature of pulmonary arterial hypertension (PAH), as heterozygous loss-of-function mutations in *BMPR2* represent the most common genetic risk factor [8,9,17]. This has focused attention on the BMP9/10–BMPR2 pathway as a key regulator of endothelial homeostasis and disease-associated dysfunction. Prior studies have demonstrated strongly context-dependent effects of BMP9/10 signaling, with both ligand administration and ligand blockade conferring benefit under different experimental conditions [37,38]. These divergent outcomes likely reflect differences in disease stage, vascular compartment, and receptor context across models, indicating that receptor context can outweigh ligand identity in determining endothelial responses.

Our data support a receptor–dosage framework in which BMPR2 expression level is a dominant determinant of how pulmonary artery endothelial cells interpret BMP9/10 signals. Using primary human pulmonary artery endothelial cells, we show that BMP9 and BMP10 uniquely activate SMAD1/5/8 signaling and promote endothelial proliferation when BMPR2 expression is intact (Figure 1 and Figure 2). An ~50% reduction in BMPR2 protein levels (an in vitro approximation of haploinsufficiency) markedly attenuates canonical signaling, abolishes proliferative responses, and is associated with a modest increase in caspase-3/7 activity (Figure 5). Conversely, increasing BMPR2 abundance enhances both SMAD1/5/8 activation and proliferation (Figure 6). Together, these findings establish BMPR2 dosage as a central constraint on BMP9/10 signaling competence in pulmonary vascular endothelium under fixed ligand conditions. While this study does not measure BMP9 or BMP10 abundance in PAH patient samples or model PAH in vivo, it defines how BMPR2 dosage constrains endothelial signaling output under controlled ligand exposure in primary human PAECs.

An important insight emerging from our data is the nonlinear relationship between SMAD1/5/8 activation and endothelial proliferative capacity. Although BMPR2 knockdown resulted in only a partial attenuation of BMP9/10-induced SMAD1/5/8 phosphorylation, this reduction was accompanied by a near-complete loss of endothelial proliferation. This mismatch is consistent with a model in which BMP9/10-driven endothelial proliferation depends on SMAD signaling exceeding a functional threshold, rather than scaling linearly with signal amplitude. While we did not titrate SMAD output against proliferative response, the loss of proliferation despite residual SMAD1/5/8 phosphorylation is consistent with a threshold-like requirement for canonical signaling. In this framework, reduced BMPR2 levels may decrease BMP9/10-driven canonical output below that required to sustain proliferation.

Context-dependent ACVR2A engagement: BMP9 and BMP10 can engage multiple type II receptors, including BMPR2, ACVR2A, and ACVR2B [32,58,59]. In PAECs with intact BMPR2 expression, BMP9/10 signaling through ALK1 is functionally BMPR2-dominant. BMP9/10 robustly activates SMAD1/5/8 and promotes proliferation, and blockade of Activin type II receptors with bimagrumab has no measurable effect despite detectable ACVR2A expression (Figure 3). Because bimagrumab targets both ACVR2A and ACVR2B, we cannot formally assign any ACVR2 contribution to a single receptor; however, ACVR2B is minimally detected in PAECs (Figure 3A), making ACVR2A the most plausible mediator when ACVR2 sensitivity emerges. More broadly, the functional selectivity of BMP9 and BMP10 over BMP7 in PAECs is consistent with established structural and biochemical studies of ALK1–type II receptor complex formation [57,58,59] and was confirmed empirically in our ligand screen (Figure 1). No new docking or computational modeling was performed in this study.

HEK293 reporter assays support this interpretation by showing that ACVR2 blockade is not sufficient to eliminate BMP10 signaling when BMPR2 is present. Bimagrumab fully suppresses Activin B–SMAD2/3 signaling yet only partially inhibits BMP10-induced SMAD1/5/8 output, leaving a substantial residual signal (Figure 4B,D; Table 1). In addition, altering type II receptor abundance shifts the balance between bimagrumab-sensitive and -resistant BMP10 signaling (Figure 4C), consistent with BMP10 output being distributed across available type II receptors.

Consistent with this distribution model, ACVR2 dependence becomes evident in PAECs only when BMPR2 is reduced. Under BMPR2-limited conditions, residual BMP9/10-induced SMAD1/5/8 phosphorylation becomes bimagrumab-sensitive (Figure 5C). Importantly, this ACVR2-supported residual signaling does not restore proliferation (Figure 5D) and coincides with increased caspase-3/7 activity (Figure 5E), indicating incomplete functional compensation rather than recovery of the BMPR2-supported program. Thus, BMPR2 dosage constrains both the amplitude and functional sufficiency of ALK1-mediated BMP9/10 signaling in PAECs. When BMPR2 is reduced to limiting levels, ACVR2A can sustain low-level output, but this output is insufficient to support proliferative responses and is accompanied by increased caspase-3/7 activity.

Reduced endothelial proliferation marks early endothelial attrition in PAH. Although advanced PAH is characterized by exuberant vascular cell proliferation, substantial experimental and clinical evidence indicates that endothelial apoptosis and impaired repair precede the emergence of hyperproliferative lesions [25,26,27,62]. In this context, the reduced endothelial proliferation observed in our system should not be interpreted as a protective brake on pathological growth. Instead, it reflects a failure of regenerative signaling capacity at the single-cell level, where BMPR2 haploinsufficiency prevents circulating BMP9/10 from sustaining endothelial maintenance. Notably, endothelial responses to BMP9 under conditions of BMPR2 loss have been reported to vary across experimental systems and degrees of receptor reduction, with proliferative responses observed at more extreme levels of BMPR2 depletion or in distinct endothelial backgrounds [33].

Consistent with this idea, we observe that BMPR2-deficient endothelial cells lose their proliferative response to BMP9/10 and show increased caspase activity, potentially capturing an early endothelial attrition state. This finding aligns with models in which initial endothelial loss promotes a permissive microenvironment for subsequent maladaptive remodeling, including smooth muscle hypertrophy and fibrosis [63]. Thus, our data help reconcile reduced endothelial proliferation with the hyperproliferative lesions of late-stage PAH by situating these processes in a temporal, two-hit model.

A two-hit model of BMPR2-dependent endothelial injury: The reduced proliferation and caspase activation observed in BMPR2-deficient endothelial cells are consistent with temporal models of PAH where early endothelial attrition and failed repair precede the emergence of hyperproliferative, apoptosis-resistant lesions [25,26,27,62]. Within this model, BMPR2 haploinsufficiency represents a first hit that compromises BMP9/10-mediated regenerative signaling, while secondary events, such as clonal selection and mural cell activation, drive advanced vascular remodeling [26,34,35,36]. We speculate that our in vitro system captures this early attrition phase but does not model subsequent clonal expansion or occlusive lesion formation, which likely require additional genetic, inflammatory, or biomechanical inputs.

Therapeutic implications: This receptor–dosage framework may help interpret context-dependent pathway modulation relevant to PAH therapeutics but does not establish a direct mechanism of action for ACVR2A-based ligand traps such as sotatercept, which has demonstrated significant benefit in PAH patients, including those with BMPR2 insufficiency [64,65]. Our data suggest that in BMPR2-deficient endothelium, BMP9/10 signaling becomes biased toward Activin type II receptor usage, resulting in impaired signaling that fails to restore proliferative capacity. In this setting, sequestration of BMP9/10, together with Activins [42,47], may limit maladaptive ligand–receptor engagement and injury-associated signaling. Ligand traps may therefore confer benefit by limiting non-productive signaling in contexts where BMPR2-dependent signaling competence is compromised.

Limitations and future directions: Experiments were performed in primary pulmonary artery endothelial cells, providing a controlled system to interrogate receptor dosage, but were limited to two independent biological samples obtained from Lonza and ATCC and do not capture the full heterogeneity of the distal pulmonary microvasculature most affected in PAH. Caspase-3/7 activity was measured as an indicator of apoptotic pathway activation. Additional orthogonal markers (e.g., Annexin V staining or TUNEL assays) would be required to confirm apoptotic commitment and distinguish early apoptotic signaling from committed cell death. Moreover, while we demonstrated altered receptor usage under BMPR2-limiting conditions, we did not interrogate non-canonical signaling pathways downstream of distinct receptor complexes. Nevertheless, these findings support a constrained, receptor–dosage-dependent signaling landscape, emphasizing loss of signaling sufficiency rather than a simple on/off receptor switch as the central pathogenic feature.

## Figures and Tables

**Table 1:** Potency of receptor traps and bimagrumab in HEK293 reporter assays.

BMP10 Inhibition By Soluble Receptor–Fc Traps (SMAD1/5/8 Reporter; BMP10 = 5 nM)												
Trap	Apparent IC_50_ (nM)				95% CI (nM)				Residual Signal (%)			
ACVR2A-Fc	186.50				152.60–225.00				0.0			
BMPR2-Fc	34.75				29.75–40.57				0.0			
ACVR2B-Fc	1.34				1.24–1.44				0.0			
Bimagrumab (BiMab) inhibition of reporter activity (BiMab = 0–300 nM)												
Ligand/ reporter	Apparent IC_50_ (nM)			95% CI (nM)			Res. signal (%)			Maximal inhibition (%)		
Activin B/SMAD2/3	3.5			2.80–4.36			8.0			92.0		
BMP10/SMAD1/5/8	63.4			23.4–unbounded			47.6			52.4		

Footnote: IC_50_ values were obtained by nonlinear regression (4-parameter logistic; X = log10 [concentration]) of normalized reporter output. RLU were background-subtracted using untreated control (UT) and normalized to the ligand-only condition (set to 100). For Bimagrumab with BMP10, inhibition is partial because Bimagrumab blocks ACVR2A/ACVR2B-mediated contributions but does not affect BMPR2-mediated signaling. The fitted Bottom reflects residual BMPR2-supported output and the IC_50_ is an apparent potency estimate for the bimagrumab-sensitive component.

## Data Availability

Datasets generated in this study are available from the corresponding author upon reasonable request. Commercial cell lines and reagents are available from their respective vendors (Lonza, ATCC, R&D Systems) as specified in the Materials and Methods. Plasmids and stable cell lines generated in this research are available from the corresponding author contingent on a material transfer agreement with Michigan State University.

## Abbreviations

The following abbreviations are used in this manuscript:

PAH	Pulmonary arterial hypertension
PAECs	Pulmonary artery endothelial cells
PASMCs	Pulmonary artery smooth muscle cells
BMP	Bone morphogenetic protein
TGF-β	Transforming Growth Factor-β
BMPR2	Bone morphogenetic protein receptor type II
ALK1	Activin receptor-like kinase 1
ACVR2	Activin type II receptor
R-SMAD	Receptor-regulated SMAD
pSMAD	Phosphorylated SMAD (active form)
SMAD	Sma/Squad Mothers Against Decapentaplegic
